# Cognitive Development and Decision Making in Basketball: A Comparison between Male Players with and without Intellectual Impairment and across Different Age-Groups

**DOI:** 10.5114/jhk/185430

**Published:** 2024-05-17

**Authors:** Javier Pinilla-Arbex, Javier Pérez-Tejero, Debbie Van Biesen, Ignacio Polo, Luc Janssens, Yves Vanlandewijck

**Affiliations:** 1 Faculty of Humanities and Social Sciences, Comillas Pontifical University, Alcobendas, Spain.; 2 “Sanitas Foundation” Chair in Inclusive Sport Studies, Faculty of Physical Activity and Sport Sciences-INEF, Universidad Politécnica de Madrid, Madrid, Spain.; 3 Faculty of Movement and Rehabilitation Science, KU Leuven, Leuven, Belgium.; 4 KU Leuven, Campus Groep T Leuven, Electrical Engineering (ESAT), TC, Leuven, Belgium.; 5 Swedish School of Sport and Health Sciences (GIH), Stockholm, Sweden.

**Keywords:** disability, performance, Paralympic, team sport

## Abstract

The aim of this study was to investigate the role of age and intellectual impairment (II) in decision-making in basketball. The current study investigated differences in decision making between equally well-trained adult basketball male players with intellectual impairment (players with II) (n = 93), adults without II (senior) (n = 44) and youth basketball players (under-14, n = 31; under-16, n = 25; under-18, n = 30). A computer test was developed composed by 20 photographs displaying various basketball game-situations, and participants had to decide as fast as possible what the player in ball possession should do: dribble, pass or shoot. Decision time and accuracy were recorded for every situation. Players with II had slower decision time (3.8 ± 1.8 s vs. 1.5 ± 0.5 s, p < 0.001) and less decision-making accuracy (15.7 ± 2.8 correct decisions vs. 17.9 ± 1.2 correct decisions, p < 0.001) compared to senior players without II. Discriminant analysis with speed and accuracy as independent variables classified 91.2% (CCA = 0.769) of the players correctly into their group: players with II or players without II. A Spearman correlation revealed that age correlated significantly (p < 0.001) with the number of correct decisions (rs = 0.269) and mean decision time (rs = −0.331). Our findings support that decision making in basketball develops with age and experience, but is significantly deteriorated in experienced adult players who have II. Decision-making should be considered as an important eligibility criterion to participate in competitive basketball events for male players with II.

## Introduction

Basketball for individuals with intellectual impairment (II) is not presently included in the Paralympic Games lineup, since athletes with II were excluded after the Sydney 2000 Games due to the incidents that occurred during those games, where some participants did not present any kind of impairment (Burns, 2015). However, basketball for athletes with II is a sport that has shown high interest in scientific literature and has a significant social impact on this population (Aksović et al., 2023).

Understanding the beneficial impact that inclusion in the Paralympics has on the growth of sports at the entry level, it is crucial for athletes with II to have the opportunity to compete at the elite level. Consequently, there is a need to establish eligibility systems grounded in research for specific sports. These systems must ensure that only those athletes with II who experience a substantial limitation in their ability to engage in a sport, such as basketball, are allowed to compete (Tweedy and Vanlandewijck, 2011). Currently, these systems do not exist for basketball. According to Mann et al. (2021), there are five steps to develop these systems: 1) identify sport and the impairment type; 2) develop the model of determinants of sport performance; 3a) develop measures of impairment; 3b) develop measures of performance; 4) assess the impairment-performance relationship; 5) determine minimum impairment criteria and class profiles. In addition, Van Biesen et al. (2021), developed a conceptual model of sport-specific classification for para-athletes with II. In this model, the evidence-based system to demonstrate eligibility of athletes with II in International Paralympic Committee sanctioned events is divided in four phases: 1) eligible impairment; 2) Generic Sport Intelligence Test (GSIT); 3) Sport-Specific Test (SST); and 4) game observation. The first two phases represent generic assessment and phases 3 and 4 are sport-specific. The present study aligns with the third phase of both models, aiming to evaluate the impact of decision-making in basketball among players with II (phase 3a of the Mann et al.’s (2021) model) using specific tests for this sport (phase 3 of the Van Biesen et al.’s (2021) model).

Basketball is a dynamic collective sport played in a constantly changing environment (Kioumourtzoglou et al., 1998; Pinilla-Arbex et al., 2016). Teammates and opponents interact at the same time with the objective to score the ball into the opponent´s basket while defending their own. These circumstances make players decide among several possibilities during the game (Gigerenzer and Goldstein, 1996; Gréhaigne et al., 2001). Players need to understand each game situation and to adapt their behavior quickly to the circumstances at any given time (Gréhaigne et al., 2001); hence, decision-making capacity is a key contributing factor to basketball.

Baker et al. (2003) defined decision-making as the ability to perceive essential information from the playing environment, correctly interpret this information and select the appropriate response accordingly. It is considered part of the cognitive area required for extraordinary preparation in high-level competitions (Blecharz et al., 2022). In basketball-related research, the study of decision-making has received considerable attention and has been developed in three main lines: 1) to analyze the cognitive components involved in the decision-making process (Araujo et al., 2009; Gorman et al., 2012; Gréhaigne et al., 2001); 2) to investigate the role of experience and the differences between experts and novices (Faubert, 2013; Gonçalves et al., 2011; Sporiš et al., 2006); and 3) to develop tools to assess decision-making capacity for talent identification (Gonçalves et al., 2011; Rösch et al., 2021).

A key question is, how cognition works during decision making? This question is of interest not only in basketball, but also in other team-sports. Gréhaigne et al. (2001) formulated an operational model for decision-making, emphasizing the importance of athletes' perception of situations and the informational resources they use to make choices. This model underscores the significance of cognitive processes and knowledge in reaching effective decisions. Conversely, Raab's approach in 2002 was anchored in the decision field theory originally developed by Busemeyer and Townsend (1993). This model adopts a dynamic viewpoint, positing that decisions emerge from a process of evaluating all the factors that argue for or against various alternatives, leading to the selection of one option over another. Contrary to this approach, Gigerenzer and Goldstein (1996) introduced the fast and frugal heuristic theory (FFH). This theory posits that decisions are often made swiftly (fast) without extensive deliberation on which choice might be best. It suggests that individuals can make quick decisions by relying on minimal information. The FFH is particularly pertinent to the sports domain, proving especially beneficial in fast-moving, dynamic sports where athletes are required to make rapid decisions, such as in basketball (Khudair et al., 2021). However, despite the differences presented in the models explained, all authors highlight that decision-making has a high cognitive implication. In addition, the literature focused also on identifying the cognitive abilities relevant in decision-making processes. Tenenbaum et al. (1993) described cognitive abilities taking part during the consecutive phases of decision making: visual strategies and attention-allocation, a selection process, anticipation, processing for making decisions, decision making elaboration, action initiation, action alteration and action evaluation. In basketball, memory retention and selective attention (Kioumourtzoglou et al., 1998), visual search and inhibitory control (Chiu et al., 2020) where identified as relevant for decision-making.

In the second research-line mentioned, multiple studies regarding team sports indicate that experience is a major predictor of successful decision-making (Tenenbaum et al., 1993) because experts develop sport-specific cognitive abilities. Expert players develop more declarative and procedural knowledge which helps them spend less time to recognize a game situation (Garland and Barry, 1991). Experienced players develop better and more accurate criteria to organize the information they receive (Gigerenzer and Goldstein, 1996) and are able to decipher relevant information from earlier display cues than novices (Abernethy and Russell, 1987). These capacities allow expert players to carry out faster and more accurate decisional processes. Additionally, expert players typically have a major repertory of motor solutions providing them with the ability to solve game situations more efficiently (Garland and Barry, 1991).

The abilities that have been identified are also linked to enhancements in perceptual and cognitive abilities. In this context, research has uncovered differences between experts and novices in areas such as visual search, anticipation (Araujo et al., 2009; Gorman et al., 2012), and learning capabilities (Faubert, 2013). Sporiš et al. (2006) observed that novices performed game actions more slowly than experts, leading to increased vulnerability to defensive pressure. More recent studies have found that inhibitory control (which is the ability to suppress inappropriate or unwanted actions) can be honed through regular exposure to complex and dynamic environments (Simonet et al., 2022), such as those encountered in basketball. This underscores the importance of factoring in basketball experience when evaluating decision-making processes (Alves et al., 2013). Furthermore, ecological domain-related tasks are key to effectively capture the complex decision-making processes acquired in real-life sport situations (Simonet et al., 2022).

The third research-line shows the complexity to assess decision-making in basketball players. Rösch and colleagues (2021) emphasized the importance of evaluating decision-making within a context that is specific to each sport, and the necessity of creating ecologically valid tasks to accurately study athletes' behavior in their particular fields of expertise. Gorman et al. (2013) employed static and moving videos to compare decision-making capacity in experts and novice basketball players, showing higher accuracy in experts´ responses. Additional research has utilized video-based tasks (Ryu et al., 2013), which revealed that skilled basketball players responded more quickly, and virtual reality settings have been employed to enhance decision-making abilities in both youth and senior players (Panchuk et al., 2018). Travassos et al. (2013) indicated that these laboratory-based approaches might not adequately represent the actual conditions of sport-specific environments, and such limitations should be considered. However, in line with Rösch et al. (2021), decision-making analysis under laboratory conditions can provide two advantages: to develop standardized methods to assess decision-making and to isolate decision-making capacity from other components that may influence decision-making during real game (e.g., self-regulation, adaptive behavior, fatigue, contextual factors, pressure); specially in population with II (Van Biesen et al., 2016).

According to the literature analyzed, to generate understanding of the cognitive processes underlying basketball performance in athletes with II (Mann et al., 2021), it seems appropriate to analyze how II impacts decision making in basketball (Burns, 2015; Sakalidis et al., 2021). Therefore, the purpose of the present study was to investigate the role of II and age on decision making speed and decision-making accuracy in basketball. This purpose was threefold: the first aim was to explore differences in decision making speed and decision-making accuracy between equally well-trained adult basketball players with and without II under testing conditions. We hypothesized that adult players with II would make slower and less accurate decisions compared to adult players without II. The second aim was to explore differences in decision making speed and decision-making accuracy between basketball players without II from different age groups, ranging from 12 years old up to the senior level. With these results we intended to draw a basketball-specific decision-making development curve. We hypothesized that speed and accuracy of decision making would improve with increasing age. The third aim was to situate the decision-making profile (speed and accuracy) of basketball players with II in the development curve of players without II. We hypothesized that players with II would demonstrate decision-making accuracy situated below the youngest age category studied of players without II.

## Methods

### Participants

A total sample of 223 male basketball players participated in this study, including one sample of a high level, adult basketball players with II (N = 93, mean age = 27.0 ± 7.0 years), one sample of adult basketball players without II (N = 44, mean age = 23.2 ± 5.1) and three samples of junior basketball players without II: under-14 years old (U-14; N = 31, mean age = 13.2 ± 1.0), under-16 years old (U-16; N = 25, mean age = 15.2 ± 0.4); under-18 years old (U-18; N = 30, mean age = 17.7 ± 0.5). Athletes with II constituted the entirety of the competitors at the World Basketball Championships held in Ankara, Turkey in 2013, and they made up 81.7% of the basketball participants at the Global Games in Guayaquil, Ecuador in 2015. The International Federation for Athletes with Intellectual Impairment (VIRTUS) was responsible for organizing these competitions. The II-basketball teams represented eight countries: Australia, France, Greece, Japan, Portugal, Poland, Turkey and Venezuela.

While the inclusion of both male and female samples is recognized as critically important in research of this nature, it is pertinent to note that the championships mentioned did not feature 5-on-5 basketball competitions for women with II due to the lack of participation of women athletes from different countries. This also occurred in other sports convened by VIRTUS. Consequently, only the male population participated in this study.

The junior and senior basketball players without II were all recruited from Belgian and Spanish competitions. To participate in the study, players were required to have at least two years of experience participating in federated basketball competitions. Every basketball player with II fulfilled the diagnostic criteria for Intellectual Disability as outlined by the American Association on Intellectual and Developmental Disabilities (Schalock et al., 2010): significant limitations in intellectual functioning (IQ ≤ 75) and in adaptive behavior, both manifested before the age of 18. Self-reported training history of all players per age group is reported in Table 1. Training history is expressed in previous involvement in basketball (years), training volume per week (hours), and total accumulated basketball experience over the lifespan (hours).

**Table 1:** Self-reported training history of all players per age group. Data presented as mean and standard deviation.

	Players with II (n = 93)	U-14 (n = 31)	U-16 (n = 25)	U-18 (n = 30)	Senior (n = 44)	Sig. *p* < 0.05
Age (years)	26.2 (6.9)	13.2 (1.0)	15.2 (0.4)	17.7 (0.5)	23.2 (5.1)	U-14 < U-16 < U-18 < Senior < II
Hours per week	6.3 (4.7)	5.9 (1.4)	5.5 (1.2)	5.4 (1.8)	4.9 (1.7)	
Months per year	9.6 (2.3)	9.7 (0.5)	9.9 (0.3)	9.8 (0.7)	9.5 (2.2)	
Playing experience (years)	9.6 (6.4)	5.48 (2.1)	4.8 (2.0)	8.6 (3.5)	14.0 (5.1)	U-14 < Senior, II U-16 < U-18, II < Senior
Total experience (hours)	2459 (2348)	1286 (631)	1041 (527)	1878 (1105)	2320 (2019)	U-14 < Senior, II U-16 < Senior, II

In Table 1, it can be seen that U-14 players show higher average values in the years they have been playing basketball and the total accumulated experience than U-16 players, since, coincidentally, it seems that this group of players started practicing basketball earlier. However, no significant differences were shown between the groups in these two variables, but there were in biological age (*p* < 0.05).

### Design and Procedures

Developing a basketball-specific decision-making test allows to isolate cognitive processing from other factors such as physical fitness, the playing position and environmental influence. Consequently, a computerized touch-screen basketball decision-making test (TS-DMT) was specifically developed for the purpose of this study by an international team of 10 basketball experts. The test consisted of 20 photographs of basketball-specific game situations which were presented to the participant on a 15.6-inch computer screen. Participants were instructed to make the quickest and most accurate decision regarding the most suitable action for the player with the ball in each given situation, as depicted in the photographs: whether to pass, shoot or dribble. The images depicting these game scenarios were sourced from official matches within Belgium's premier basketball division, the “Pro-league”. In each scenario, the shot-clock was clearly visible to participants, and the location of the ball was emphasized.

Three university level basketball experts, each with over 15 years of experience in teaching and coaching at the university level, initially selected the game situations, determined the shot clock times, and agreed upon the correct decisions for each scenario. In line with protocols employed in previous studies to assess decision making from a sample of real-game situations (Sabag et al., 2023), a panel of seven experts, each having over 20 years of basketball experience, refined and finalized this initial selection. For each photograph depicting a game situation, the experts were tasked with identifying the optimal decision that the player in possession of the ball should make. The experts presented a high level of agreement (ICC = 0.94) when deciding on the correct response for each given situation. The specific software for the TS-DMT administration was developed by engineer L.J. The software featured a menu with four buttons designed to access the participant's personal data and to initiate the demonstration trial (N = 1), the practice trials (N = 4), and the actual test trials (N = 20).

During the actual test, each game situation was displayed in a uniform manner: initially, a shot-clock was shown at the top of the screen to indicate the remaining time for ball possession. Following a two-second interval, the image of the game situation appeared, with the position of the ball marked by a yellow circle that vanished after one second. Once the participant decided the action to take, they were required to press the space bar. This action triggered the appearance of a new screen with three pictograms, each symbolizing the options to shoot, dribble or pass, from which the participant could then make their selection. The participant touched the respective action button on the screen to indicate his decision. When the participant indicated to be ready, the experimenter initiated the next game situation.

The test was administered in a quiet room, free from any distractions. Participants were seated in front of a 15.6-inch touch-screen computer (ASUS All-in-one EeeTop ET1611DUT). First, personal data from the player was gathered including: name, surname, birth date, country, team, hand dominance, playing position and test administrator. Next, participants were presented with a step-by-step explanation and demonstration of the test. The final part of the introduction consisted of a trial with four game situations to ensure that the participant correctly understood how the test functioned. The actual test consisted of presenting the 20 game situations in a standardized sequence with an increasing difficulty level. All test administrators were trained to conduct the test in a standardized way.

### Measures

The variables recorded for each game situation presented in the test were decision-making accuracy (binary outcome: correct decision or not), and decision-making time (ms). Decision-making time was calculated automatically by computer software from the moment in which the photograph appeared on the screen until the player pressed the space bar.

### Statistical Analysis

To check that the sample employed was over the minimum sample size required to test the study hypotheses, a power analysis was conducted using G*Power version 3.1.9.7 (Faul et al., 2007).

The Kolmogorov-Smirnov test was used to assess the normality of the data. Descriptive statistics, specifically the mean and standard deviation, were computed for both players with and without II across each sub-sample. These statistics were calculated for the variables of decision speed (the average time taken to make a decision, measured in ms) and decision accuracy (the total number of correct decisions out of a maximum of 20). To evaluate the first hypothesis, an independent samples *t*-test was initially performed to compare the differences in decision time and decision accuracy between senior players with II and those without II.

Furthermore, to discern which variables most effectively differentiated players with II from those without, a discriminant analysis was performed. The structural coefficients (SCs) derived from the discriminant function were utilized to pinpoint these variables. A structural coefficient with an absolute value greater than │0.30│ was considered significant for distinguishing between the two groups, as per Ntoumanis (2003). To validate the discriminant models, a leave-one-out classification method was applied, also recommended by Ntoumanis (2003).

To address the second hypothesis, a one-way ANOVA followed by a post hoc Tukey test were used to compare the results of the test across different sub-samples of players without II. Additionally, a Spearman correlation coefficient was calculated to assess the relationship among the age group, the accuracy and speed of decision-making.

To test hypothesis three, a general linear model with a fixed factor group was performed, using Tamhane post hoc, as variance between groups was found not homogenous. Also, discriminant analysis with mean time and correct decisions during TS-DMT (speed and accuracy) as independent variables was performed, in order to assess where the decision-making capacity of adult basketball players with II was situated within the developmental curve of youth basketball players from different age groups. All statistical analyses were performed using PASW statistics 20 (SPSS Inc., Chicago, IL, USA). Statistical significance was set at *p* < 0.05.

## Results

The values obtained from the power analysis for each hypothesis ranged between 0.941 and 1.0, with the effect size set at 0.8 and significance at *p* < 0.05. These results were high, thereby reducing the risk of committing a Type II error (Faul et al., 2007).

Players with II decided significantly slower (3.6 ± 1.4 s per photograph) and less accurately (15.9 ± 2.3 correct decisions in the whole test) compared to their age-matched group: the Senior players group (1.5 ± 0.5 s and 17.9 ± 1.2 correct decisions, *p* < 0.001; hypothesis one). To assess the differences in decision time and accuracy across age groups (hypothesis two), one-way ANOVA revealed significant differences among the sub-samples of players without II, who were grouped by age, in terms of the variables mean decision time (F3 = 4.3; *p* < 0.05) and the number of correct decisions (F3 = 5.4; *p* < 0.05). The specific test outcomes for each group, along with the differences identified between groups via post hoc Tukey test, are detailed in Table 2.

**Table 2:** Test outcomes per group and differences detected between groups.

	Players with II	U-14	U-16	U-18	Senior	*p* < 0.05
Mean Time	3.6 (1.4)	2.0 (0.7)	2.0 (0.9)	2.1 (1.3)	1.5 (0.5)	Senior < U-14, U-16, U-18, players with II
Correct	15.9 (2.3)	16.5 (2.2)	17.3 (1.8)	17.7 (1.4)	17.9 (1.2)	Senior, U-18 > U-14, players with II

**Figure 1:**
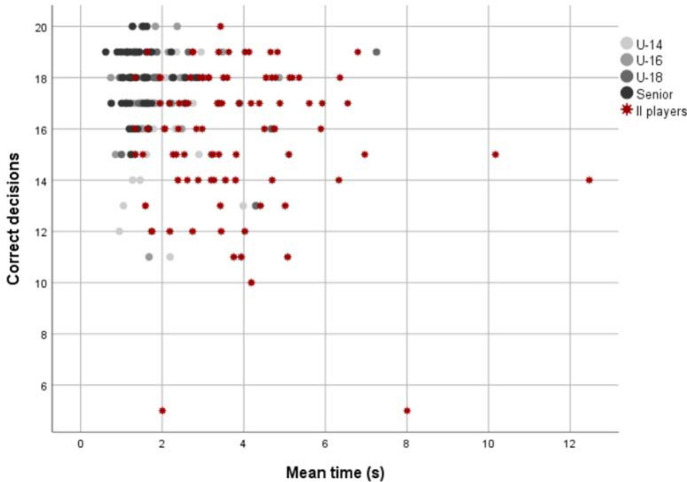
Distribution of players' results (time and correct decisions) per group.

Distribution of players' results (time and correct decisions) per group is visualized in Figure 1; players with II show more variability, and their results tend to be in the right-lower part, while the results of players without II tend to be in the left-upper part. However, there is an overlap between some of players without II (especially those from the U-14 group) and players with II.

Although no anomalies were observed during the administration of the tests, four players with II performed markedly below the rest of the players with II (Figure 1). The data from these four players were not included in statistical analyses as they were considered outliers.

The discriminant function that was computed included both decision time and decision accuracy as variables, taking into account all senior players in the analysis (with and without II) in each game situation, except situation 13, which was considered an outlier and removed from the data (situation 13 had less than 36% correct responses). The discriminant function was found to be statistically significant (*p* < 0.001), which suggests a strong predictive relationship with a canonical correlation of 0.802. The discriminant function that was calculated is presented below:

D = – 0.131Dt1 + 0.138Dc1 – 0.083Dt2 + 0.579Dc2 + 0.102Dt3 + 0.893Dc3 – 0.036Dt4 + 0.061Dc4 + 0.1Dt5 – 0.001Dc5 + 0.03Dt6 + 0.062Dc6 – 0.149Dt7 + 0.362Dc7 – 0.057Dt8 + 0.384Dc8 – 0.231Dt9 – 0.426Dc9 + 0.079Dt10 + 0.335Dc10 – 0.166Dt11 – 0.632Dc11 + 0.045Dt12 + 1.094Dc12 – 0.103Dt14 + 2.218Dc14 – 0.096Dt15 – 0.244Dc15 + 0.127Dt16 – 0.231Dt17 + 0.387Dc17 – 0.102Dt18 + 0.053Dc18 – 0.031Dt19 + 1.044Dc19 + 0.119Dt20 + 0.776Dc20 – 3.667

where Dtn = decision time in photograph “n”; Dcn = accuracy of the decision in photograph “n” (0 = incorrect, 1 = correct).

When results from each player were introduced into this function, a “D value” was obtained. This value represents the proximity of the player´s outcomes in the test with the reference values from players with and without II. When the calculated “D value” was higher than 0.479, the player was classified as a “player without II” and, when it was lower, the player was classified as a “player with II”. This function classified 91.0% of the players correctly according to the diagnostic group (players with II or players without II).

The structural coefficients showed that the time taken to make a decision in each photograph (with the exception of photograph 19) was the variable that significantly contributed to the discrimination between players with II and those without, with a structural coefficient (SC) of │0.30│ or higher. A Spearman correlation analysis revealed significant relationships between the age group and the number of correct decisions (r = 0.269, *p* < 0.05), as well as between the age group and the average time taken to make a decision for each photograph (r = −0.331, *p* < 0.001).

**Figure 2:**
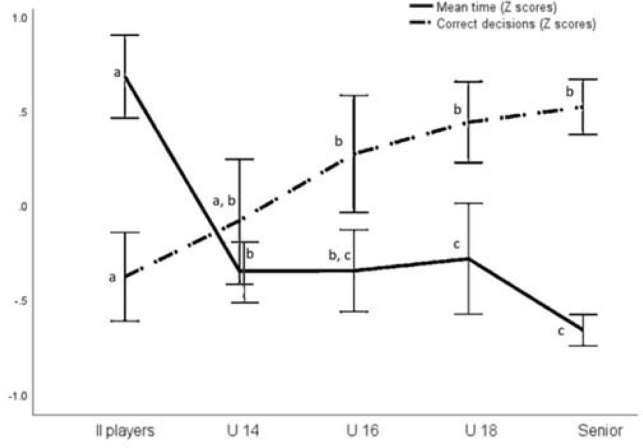
Mean time and correct decisions performed by each group in the test.

To position the performance of players with II relative to the various sub-groups of players without II (as per hypothesis three), Figure 2 presents a comparison between groups´ outcomes in the test, using Z scores as standardized data from variables. Differences were found between the different age groups with the general linear model (with post hoc Tamhane). As depicted in Figure 2, the outcomes from players with II were below the results of U-14 players. Players with II spent significantly more time than every sub-sample of players without II (*p* < 0.05) and they made less accurate decisions compared to all the sub-samples except the sample of U-14 players (*p* < 0.05).

Regarding players´ hand dominance, no significant differences (*p* > 0.05) were observed between right-handed and left-handed athletes within each sample.

## Discussion

The aim of this study was to explore the impact of II and age on the speed and accuracy of decision-making in basketball. In line with predictions, it was found that adult basketball players with II took longer and made less accurate decisions than their counterparts without impairment who were of similar age, had comparable experience, and engaged in an equivalent amount of training. Secondly, the results confirmed the progressive improvement in accuracy of decision-making and speed of decision making with increasing age in young basketball players without II. Finally, the decision-making speed of adult players with II appeared to be situated below the performance of players in the youngest age category of players without II that participated in our study (i.e., 13 year old players).

As it seems from our results, cognition plays a dominant role in these decision-making basketball related tasks, with players with II having similar age and training volume scoring below the youngest age category (Figure 2), thus it seems that, for these players, their age and experience could not compensate sufficiently for their cognitive impairment in these basketball related tasks. These results are in line with the study by Pinilla-Arbex et al. (2016), where decision-making speed and accuracy were investigated during standardized game-situations on a basketball court in two samples of well-trained players with and without II. Basketball players in that study had to make the correct decision while performing the corresponding basketball action on the court, with speed and accuracy. Players with II in the field-study scored less points (successful shots) resulting from their actions, and they required more time to decide and execute the decision. Interestingly, no differences were found in the number of correct decisions. This finding is different compared to the present study, using a computerized test to assess decision making, in which players with II required more time and performed less accurately compared to players without II. Despite the obvious difference in assessment between both studies (on the court versus computerized), there were other aspects that might have influenced the observed differences (e.g., total number of basketball situations, difficulty of the presented situations, 3 n 3 field versus 5 on 5 computerized situation).

Using a computerized assessment of decision-making is useful to enhance standardization and measure accurately (Rösch et al., 2021). The decision-making development curve (Figure 2) showed that results improved progressively across the different age-categories, suggesting that the cognitive processes assessed in this test are relevant for basketball expertise development, demonstrating validity of the instrument. However, players with II might have a limited capacity to transfer one situation (computer test) to another one (real game, Travassos et al., 2013), which may also contribute to the differences in outcomes between the two studies (Alves et al., 2013; Page et al., 2019). Nevertheless, future studies may analyze the relationship between the results in the TS-DMT test and basketball performance in real games.

Regarding the decisional speed, there is consensus in the literature that it is a key determinant for basketball success (Araujo et al., 2009). Consequently, II might have a negative influence on basketball performance. Other studies, based on the opinions of coaches with expertise in working with players with II, indicate that basketball players with II seem to present limitations mainly in the tactical components of basketball (Polo et al., 2017). This observation might be partially explained by the influence of II on decisional speed, as also significant differences were found when comparing the different sub-samples of players without impairment grouped in different age-groups. The positive relationship found among age, experience and decision making in this study is in line with previous studies (Kioumourtzoglou et al., 1998; Tenenbaum, 2003). As Tenenbaum et al. (1993) indicated, experience has been attributed as the major predictor of successful decision-making and seems to confirm the implication of decision making in basketball performance. In this line, the study by Iglesias et al. (2005) demonstrated that knowledge exercised in the performance of some tasks was related to performance and experience; this fact may explain the positive relationship found in our study between decision-making accuracy and experience.

At this point, the crucial question emerging is to what extent the improvement in decision making ability across the different age-groups is explained by experience, cognitive development or training? Popovic et al. (2014) found that athletes improved their cognitive abilities depending on the sport they practiced. In that study, the conclusion was drawn that track-and-field athletes exhibited superior symbolic reasoning skills, meaning they had a greater understanding of verbal content, compared to basketball players. However, basketball players demonstrated a more pronounced capacity for abstraction and generalization processes, as well as an enhanced ability to receive and process information. It seems that athletes reach some kind of “cognitive specialization” by improving the cognitive abilities that best contribute to sport-specific performance. This may explain the way athletes mature and increase their experience: they improve these cognitive abilities. The results obtained across the different age-groups regarding decision making capacity can be supported by the development of their cognitive abilities. If this idea proved to be correct, it could mean that athletes with II are limited in their ability to reach such a level of specialization. However, more research is needed to understand the mechanisms underlying decision-making development in youth players.

Results from the present study have the potential to orient future research. Although the negative influence of II on decision-making was observed, it remains unknown to what extent the lower decisional speed presented by athletes with II is due to the influence of II on the perceptive or decisional processes (Gréhaigne and Godbout, 1995). Further studies might compare gaze control between players with and without II, or how they discriminate between the relevant stimuli. Also, it would be beneficial to investigate and identify the cognitive abilities that may determine the specific decisional phase of decision making, or how these abilities are developed according to the players´ age.

One of the contributions of the present study is the test design. The computer test was designed to be applicable for a population with II and for different languages and cultures. This allows the test to be applicable to diverse samples independent of their age or capacities, worldwide. In addition, the use of a computerized test allows to focus on the analysis of the cognitive component of decision making, avoiding the effects of physical skills or contextual components that could take part during real game-situations (Rösch et al., 2021; Van Biesen et al., 2016). Also, results showed a potential ceiling effect providing the potential barriers that trained II-basketball players can have in decision-making. Although the pictures used in the test were not classified by the level of difficulty, the calculated discriminant function showed that there were pictures with more weigh to discriminate between players with and without II (e.g., picture 7 or 14). These pictures might represent game-situations where players with II had more difficulties to decide successfully.

Additionally, the results from this study could be used as reference values for future studies investigating decision-making in basketball. Hence, the computerized decision-making test could be a potential tool for early detection of basketball talent in terms of cognitive capacities for players with and without II, a question that was of interest in previous research (Burns, 2015).

Lastly, by employing the calculated discriminant functions, we can identify those well-trained athletes who exhibit significant limitations in decision-making in basketball, which is crucial for the development of evidence-based eligibility systems. According to the model proposed by Mann et al. (2021), this study developed a new measure of impairment (phase 3a of the model) based on decision-making capacity in basketball. Furthermore, through the discriminant function, it is possible to establish a minimum impairment criterion (phase 5 of the model). Additionally, the test used in this study constitutes a sport-specific assessment under testing conditions, fulfilling the requirements of the model for sport-specific classification of para-athletes with II, as outlined by Van Biesen et al. (2021).

However, further research is needed to address certain questions in the development of eligibility systems for basketball athletes with II. These include assessing the relationship between the test results and performance under actual game conditions (phase 4 of the Mann et al.’s (2021) model), determining how to detect misrepresentation during the tests, and developing systems for game observation, as suggested by Van Biesen et al. (2021).

Some of the strengths of this study include a large sample size (power analysis over 0.9), a diverse population resulting from the inclusion of players with II and players without II across different age-groups and the novelty of the approach. However, a limitation of this study was the exclusion of a tapping test or a similar test, to assess upper limb motor control in participants. Although no athlete reported any kind of physical impairment and previous studies have not found limitations on motor skills when comparing players with II and players without II (Van Biesen et al., 2016), controlling this variable might detect the influence of motor skills on the test results. Another limitation is the lack of female participation in this study, highlighting the necessity for further research in the female population to determine whether the findings are consistent with or differ from those observed in this study.

## Conclusions

This study contributed to our understanding of how cognition and decision making in basketball are related; this was achieved by means of a basketball-specific decision-making task assessing decision-making speed and accuracy. Senior basketball players with II performed below the level of 12- to 14-year-old basketball players without II. However, we still do not know the exact mechanisms underlying this relationship, thus further research is needed. The results of this study may be helpful when developing basketball eligibility systems for players with II. They can help identify and ensure that only athletes with significant basketball-specific cognitive limitations, such as decision-making limitations, participate in II-competitions.
